# Field testing of a lightweight, inexpensive, and customisable 3D-printed mosquito light trap in the UK

**DOI:** 10.1038/s41598-019-47511-y

**Published:** 2019-08-06

**Authors:** Tomonori Hoshi, Victor A. Brugman, Shigeharu Sato, Thomas Ant, Bumpei Tojo, Gaku Masuda, Satoshi Kaneko, Kazuhiko Moji, Jolyon M. Medlock, James G. Logan

**Affiliations:** 1 London School of Hygiene and Tropical Medicine, Keppel Street, London, WC1E 7HT United Kingdom; 2 Department of Eco-Epidemiology, Institute of Tropical Medicine, Nagasaki University, Nagasaki, 852-8523 Japan; 3 School of Tropical Medicine and Global Health, Nagasaki University, Nagasaki, 852-8523 Japan; 4 Vecotech Ltd, Keppel Street, London, WC1E 7HT United Kingdom; 5 Faculty of Medicine and Health Sciences, University Malaysia Sabah, Sabah, 88400 Malaysia; 6 Public Health England, Porton Down, Salisbury, SP4 0JG United Kingdom

**Keywords:** Ecology, Malaria

## Abstract

Mosquito surveillance is a fundamental component of planning and evaluating vector control programmes. However, logistical and cost barriers can hinder the implementation of surveillance, particularly in vector-borne disease-endemic areas and in outbreak scenarios in remote areas where the need is often most urgent. The increasing availability and reduced cost of 3D printing technology offers an innovative approach to overcoming these challenges. In this study, we assessed the field performance of a novel, lightweight 3D-printed mosquito light trap baited with carbon dioxide (CO_2_) in comparison with two gold-standard traps, the Centers for Disease Control and Prevention (CDC) light trap baited with CO_2_, and the BG Sentinel 2 trap with BG-Lure and CO_2_. Traps were run for 12 nights in a Latin square design at Rainham Marshes, Essex, UK in September 2018. The 3D-printed trap showed equivalent catch rates to the two commercially available traps. The 3D-printed trap designs are distributed free of charge in this article with the aim of assisting entomological field studies across the world.

## Introduction

Mosquito surveillance is fundamental to the monitoring of vector-borne disease risk and the planning and evaluation of vector-control strategies. A wide variety of different sampling methods are used^1^, among which the United States Centers for Disease Control and Prevention (CDC) light trap, a battery-operated portable light trap, is extensively used for the collection of a wide range of mosquito species. Key species include members of the genus *Anopheles* and, accordingly, many organizations use these traps as a core part of their vector surveillance programs^2–4^. The BG-Sentinel trap (Biogents, Germany) with accompanying attractant lure (BG-lure) is also frequently used to sample a wide range of mosquito species during both the day and night, notably *Aedes* and *Culex* species^5^. Together with others, these traps provide effective and rapidly deployable mosquito trapping capabilities. However, for some organisations and governments, the trap and shipping costs can be prohibitive for large-scale deployment.

In recent years, the availability of 3D printing technology or additive manufacturing, involving the creation of a physical object from digital modelling data, has dramatically increased^6–9^. The 3D printer gradually stacks layers of the chosen material such as polylactic acid (PLA) filaments to build up the digitally modelled 3D objects. The biggest advantage of the 3D printer is that anybody in the world who has access to a 3D printer or 3D printing services can create uniform objects when 3D model data are provided. Although the design, creation and modification of the 3D object require a computer and specialist software, several user-friendly software programs suitable for beginners are available for free. These include, but are not limited to, Design Spark Mechanical (https://www.rs-online.com) and FreeCAD (https://www.freecadweb.org). The size and cost of 3D printers has greatly decreased in the past few years due to expired core patents related to the 3D printing technology and compact benchtop devices are now widely available. These technological developments mean that an organisation would only require a one-time purchase of a 3D printer setup and, with the availability of an appropriate digital model, could produce multiple traps at a low cost per trap.

Here, we used 3D printing technology to design a light trap with minimal production costs using a freely-available design software. The trap was designed to have a reduced weight and size compared to most commercially available models whilst maintaining equivalent trapping efficiency. Furthermore, the trap was designed to reduce the required battery weights, often the heaviest component of a trapping setup, thus enhancing portability. In this study, we compared the sampling efficacy of our 3D-printed mosquito light trap with the CDC light trap and BG-Sentinel 2 trap in Rainham Marshes, Essex, United Kingdom (UK). This is a managed area of grazing marshland in the Thames Estuary region which supports abundant populations of wildlife and is situated close to both London and to areas of similar marshland habitat where large mosquito populations have been described^10^. Moreover, Rainham Marshes is a site targeted as part of routine mosquito surveillance by Public Health England due to possible risk of incursion of invasive mosquito species and exotic pathogens. Here we present results of the trap comparison study and discuss the potential for wider adoption of 3D-printing into trap design and the incorporation of the 3D-printed light trap into vector surveillance programs.

## Results

**Figure 1:**
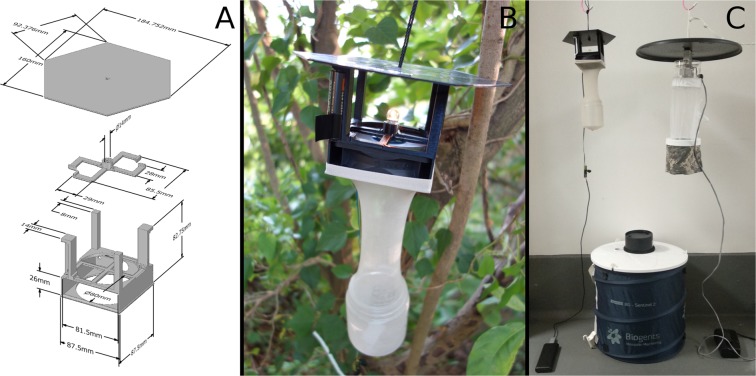
The panels show: (**A**) the 3D-printed trap blueprint comprising three pieces; (**B**) the 3D-printed trap in field operation; and (**C**) trap size comparisons between the 3D-printed trap, CDC light trap, and BG-Sentinel 2 trap.

The 3D-printed trap design was successfully produced with a reduced size and weight compared to the two commercial traps (Fig. 1 & Table 1). Excluding battery weight, the 3D-printed trap weighs 238 g which is 553 g and 977 g lighter than CDC light trap and BG-Sentinel 2 trap, respectively (Table 1). Furthermore, the total cost of the 3D-printed trap in the UK is approximately $12.46 USD (range $11.76–13.22 USD), which is $139.54 USD (91.2%) lower than that of a CDC light trap and $194.24 USD lower (93.97%) than a BG Sentinel 2 trap. Including the initial upfront investment costs of the 3D printer setup and batteries, the total cost for ten 3D-printed traps stands at $906.18 USD, a 62.53% and 63.59% reduction in costs as compared to ten CDC light traps ($2,418.63 USD) or BG-Sentinel 2 traps ($2,488.65 USD), respectively.

**Table 1:** Comparison of costs between a standard setup of the three trap types used in this study.

	3D-printed trap		CDC light trap		BG Sentinel 2 trap
Manufacturer	The end-user		John W. Hock, USA		Biogents AR, Germany
Initial capital investment	3D printer: $200–290		$0		$0
**Cost per trap body**					
*in the UK*	$11.76–13.22 (£9.04–10.17)		$152 (£116.95)		$206.7 (£159.00)
*in Japan*	$9.12–16.63 (¥1,140–2,079)		$302.4 (¥37,800)		$478.4 (¥59,800)
*in the US*	$10.11–12.97		$106		$204
*in Malaysia*	$15.80–16.05 (MYR63.20–64.21)		$188.25 (MYR753)		$425 (MYR1,700)
Trap weight	238 g		771 g		1,215 g
Rechargeable battery	One portable battery	Two AA batteries	Four D batteries	[One 6 V motorbike battery]	One 12 V motorbike/car battery
*Cost*	$28.59–42.89	$3.16–4.84	$32.49–67.03	[$14.98–58.37]	$24.66–56.50
*Weight*	181–365 g	54–62 g	620–660 g	[870–2,260 g]	2,200–6,100 g
*Running time*	15–25 h	14–20 h	[24–40 h]	17–48 h	
*Battery charger*	$1.70–3.08	$1.94–5.84	$20.79–22.74	[$23.39–38.99]	$19.49–38.99
Average overall costs for 1 trap	$379.85		$241.86	[$219.86]	$276.52
Average overall costs for 2 traps	$438.09		$483.73	[$439.72]	$553.03
Average overall costs for 10 traps	$906.18		$2,418.63	[$2,198.60]	$2,488.65
Operational strengths	Lowest cost per trap of the three. Portable batteries enable reduced battery size and weight compared to 6 V and 12 V batteries. Easy to print replacement parts for repair. Users can modify the design for their own purposes using free CAD software. Suitable materials for printing can be selected for each study environment.		It is easier and quicker to turn on/off the trap than other two traps. The collection box is well-designed and feasible to set up and retrieve. The lights can be helpful to see if the trap is running from a distance.		Mosquito samples do not pass through the fan and so are less damaged. The trap has a well designed recess for installation of an attractant such as the BG Lure and an optional CO2 gas setup. The trap can be operated using either a battery in remote areas or AC power supply, theoretically allowing a 24 hour/365 day operation.
Operational concerns	Users may require training in the use of CAD software and 3D printer use. Creating trap pieces takes about 12 hours, which could be an issue where electricity supplies are unstable. Some of the filament types are weak against a high temperature (>60–70 °C) thus direct sunlight may deform the trap. Some materials might be difficult to purchase in remote areas in Africa and Asia. Electronic circuits for the light could be unstable. The trap is lightweight and so may be adversely affected by strong winds.		Faults with the electronic wiring can occur, and there is no protective structure. The trap is cumbersome to transport and carry in the field and is difficult to repair. Heavy 6 V batteries (or 12 V with optional adaptor) required.		When removing the catch bag from the intake funnel the captured mosquito samples can be damaged. The usual placement of the trap on the floor opens it up to possible damage from animals or the wind if not secured. Carrying mosquitoes in the sampling bag without damaging them is a challenge. Heavy 12 V batteries required if not plugged in.
Additional challenges for all sampling methods	CO_2_ baits produced using the yeast-mixture setups can be heavy to transport and need to be replaced daily. However, this approach is still superior to the use of gas canisters or dry ice in remote areas.				
	Predacious insects (e.g. spiders, ants) can enter into the traps and consume the catch.				

This does not include the costs of the method of CO_2_ production or additional attractants. Prices correct as of January 2019. The costs were estimated based on online shopping sites (e.g. Amazon, AliExpress, and Shopee) and local shops. Converted prices into US dollar are shown along with the original currency in each country, if available. The conversion rates are: £1 (GBP) = $1.3 (USD); ¥1 (JPY) = $0.008 (USD); and MYR1 (Malaysian Ringgit) = $0.25 (USD). Average overall costs were based on the best materials and equipment available in the UK.

A total of 1154 mosquitoes was collected, comprising eight species and four genera (Table 2). The 3D-printed trap captured the most mosquitoes, collecting a total of 532, followed by the CDC light trap and BG-Sentinel 2 traps (310 and 311, respectively). The dominant two species across the sampling methods were *Culiseta* (*Culicella*) *morsitans* (Theobald 1901) (866 samples, 75.0%) and *Culex pipiens s.l*. (238, 20.6%). The remaining six species totalled fewer than 10 samples, except for 25 samples of *Anopheles* (*Anopheles*) *claviger s.s*. (Meigen 1804). No statistical significance was observed between the total and species-sex stratified numbers of mosquitoes (see Supplementary Fig. S1).

**Table 2:** Total (mean ± SD) and species- and sex- stratified numbers of mosquitoes collected over 12 nights by each of the three sampling methods.

	3D-printed trap		CDC light trap		BG-Sentinel 2 trap		
Species-sex	n	(mean ± SD)	n	(mean ± SD)	n	(mean ± SD)	*P* value
*Culiseta* (*Culicella*) *morsitans* (Theobald 1901)	**400**	**(16.67 ± 27.81)**	**213**	**(8.88 ± 13.85)**	**253**	**(10.54 ± 14)**	**0.62**
Female	400	(16.67 ± 27.81)	213	(8.88 ± 13.85)	253	(10.54 ± 14)	0.62
Male	0	—	0	—	0	—	—
*Culex pipiens s./*.	**118**	**(4.92 ± 5.38)**	**69**	**(2.88 ± 3.47)**	**51**	**(2.12 ± 2.38)**	**0.16**
Female	117	(4.88 ± 5.38)	62	(2.58 ± 3.43)	51	(2.12 ± 2.38)	0.12
Male	1	(0.04 ± 0.2)	7	(0.29 ± 0.75)	0	—	0.061
*Culex (Barraudius) modestus* Ficalbi 1889	**1**	**(0.04 ± 0.2)**	**0**	**—**	**0**	**—**	**0.37**
Female	1	(0.04 ± 0.2)	0	—	0	—	0.37
Male	0	—	0	—	0	—	—
*Culiseta* (*Culiseta*) *annulata* (Schrank 1776)	**1**	**(0.04 ± 0.2)**	**3**	**(0.12 ± 0.45)**	**3**	**(0.12 ± 0.34)**	**0.59**
Female	0	—	1	(0.04 ± 0.2)	3	(0.12 ± 0.34)	0.16
Male	1	(0.04 ± 0.2)	2	(0.08 ± 0.41)	0	—	0.60
*Anopheles* (*Anopheles*) *maculipennis s.l*. Meigen 1818	**1**	**(0.04 ± 0.2)**	**6**	**(0.25 ± 0.9)**	**0**	**—**	**0.35**
Female	0	—	3	(0.12 ± 0.45)	0	—	0.13
Male	1	(0.04 ± 0.2)	3	(0.12 ± 0.45)	0	—	0.35
*Anopheles* (*Anopheles*) *claviger s.s*. (Meigen 1804)	**7**	**(0.29 ± 0.75)**	**18**	**(0.75 ± 2.07)**	**0**	**—**	**0.071**
Female	7	(0.29 ± 0.75)	13	(0.54 ± 1.41)	0	—	0.072
Male	0	—	5	(0.21 ± 0.83)	0	—	0.13
*Anopheles* (*Anopheles*) *plumbeus* Stephens 1828	**2**	**(0.08 ± 0.41)**	**0**	**—**	**2**	**(0.08 ± 0.41)**	**0.60**
Female	2	(0.08 ± 0.41)	0	—	2	(0.08 ± 0.41)	0.60
Male	0	—	0	—	0	—	—
*Coquillettidia* (*Coquillettidia*) *richiardii* (Ficalbi 1889)	**2**	**(0.08 ± 0.28)**	**3**	**(0.12 ± 0.34)**	**1**	**(0.04 ± 0.2)**	**0.58**
Female	2	(0.08 ± 0.28)	3	(0.12 ± 0.34)	1	(0.04 ± 0.2)	0.58
Male	0	—	0	—	0	—	—
Total samples	532		312		310		0.56
Total spp.	8		7		4		

## Discussion

This study aimed to create and test a 3D-printed mosquito light trap for use in field collections of mosquitoes. The 3D-printed trap design was successfully produced at a low total cost of below $20, excluding the battery. Although the average 3D printer setup requires an initial investment not required for the commercially available traps (Table 1), this approach rapidly becomes cost-effective and offers long-term cost savings, particularly when considering the minimal cost of printing replacement parts. For example in the UK, the initial investment of purchasing one 3D printer (our printer cost £205 GBP) can be recouped when more than two 3D-printed traps are used instead of two CDC light traps or BG-Sentinel 2 traps in a surveillance programme.

The 3D-printed trap is lightweight, can easily be taken apart for transportation and is quick to assemble upon arrival at a field site. Results indicate that the 3D-printed trap with CO_2_ bait collects mosquitoes in the field with a comparable sampling efficacy to the CO_2_ baited CDC light trap and BG Sentinel 2 trap with CO_2_ and BG-Lure attractants, two gold-standard traps in widespread usage worldwide. Collectively, these results demonstrate the exciting possibility of rapidly increasing the availability of traps in geographically remote and resource-poor locations using 3D printing technology. In theory, anybody can create and repair the 3D-printed trap when a 3D printer is available after a short initial training period at a far lower cost in the long-term, offering improved return per dollar of funding.

The current CDC light trap is a well-designed trap that has been proven to show consistent sampling results in many different environmental settings across the world^11,12^. The BG Sentinel 2 trap is a lightweight, highly portable trap widely used for the surveillance of invasive *Aedes* species, particularly *Ae. aegypti* (Linnaeus 1762) and *Ae. albopictus* (Skuse 1894), for which it has repeatedly proven its high sampling efficacy^13,14^. Although these traps were designed to be lighter and less bulky than earlier mosquito trap designs^15^, we experienced the drawbacks of battery weight during the fieldwork (Table 1).

In designing the 3D-printed trap with lightweight materials and enabling its powering by widely-available portable power banks (such as those used to charge mobile phones), we have overcome the major weight limitations of some current trap setups. These portable battery packs are lightweight, easily recharged and of lesser or equivalent cost to the 6 V (or D cell) and 12 V traps used for the commercial traps. The AA rechargeable batteries used to power the light are also cheap and readily available. Together with the cost benefits the 3D-printed trap clearly offers significant advantages for field collectors.

Despite these benefits there are important considerations to take into account prior to the wider deployment of the traps. Firstly, the trap has only been tested at a single field site and so conclusions are limited to the species complement present there and at the time of year selected for the study, which is towards the end of the peak mosquito period in the United Kingdom^16^. In addition, this study used a yeast mixture to generate CO_2_ to attract mosquitoes, which may provide a more variable or lower overall CO_2_ release rate than dry ice. Furthermore, the trap has not been tested for its efficacy in trapping invasive *Aedes* or *Anopheles* species, an important component of surveillance systems in many parts of the world. No invasive species are considered to be established in the UK despite recent detections^17,18^. Further studies in the UK and further afield are underway to address these data gaps but we envisage a collaborative approach to gathering further data on trap performance for collecting different species to allow for optimisation. This includes testing the trap with different attractant blends or visual cues relevant to the target mosquito species in general. The flexibility of the 3D design allows for such modifications to be made with relative ease.

There are several additional practical limitations to the 3D-printed trap design at present. One issue was that the aluminium tape comprising the battery contact points of the electronic circuit wore out on occasion. To mitigate this, aluminium foil sheets were used to fill the gaps to complete the circuit. Selecting a more robust, but equally low-cost material may be one way of overcoming this issue. A wide range of materials suitable for various applications are available from many sources at a reasonable price. In the future, we believe that 3D printers will be able to create a trap with integrated printed electronic circuits which would preclude such issues.

Secondly, the quality of 3D-printed objects is highly dependent on printer settings and filament materials. Some budget-range 3D printers and filaments may not be able to produce each piece of the trap with full precision as modelled in the CAD software or with enough durability for fieldwork. On occasion the surfaces of the printed pieces had to be sanded down to allow for an exact fit. As the quality of 3D printers and filament materials continues to improve we envisage this issue rapidly disappearing.

Finally, some additional structural customisations to the design could be made for various purposes such as adding a pocket in which a lure could be placed or to include fittings for a different light source such as coloured or ultraviolet light emitting diodes (UV-LEDs) for collecting different vector groups such as *Culicoides*^19^. By openly disseminating the method of trap design and the designs themselves we believe that researchers working in different settings will be able to modify the trap designs to suit their aims (see Supplementary Data 1). With the low costs of this approach, we hope that both professional and amateur researchers will be able to contribute to the optimisation and testing of the 3D-printed trap in different settings worldwide. We would particularly advocate the incorporation of this trap into citizen science projects. In this paper, two versions of the trap CAD model are available: the tested version (see Supplementary Fig. S2 and Data 2) and an improved version (see Supplementary Fig. S3 and Data 3). The improved version has three changes: (1) an improved structure to connect the rain shield and main trap body pieces to increase robustness; (2) an enlarged outlet size in the main trap body for improved airflow; and (3) a frame has been added to the collection bag section to provide more structure to the soft material (e.g. nylon stockings) and thus improve the chances of collecting intact specimens. In addition, this section could be used to keep captured mosquitoes alive for further experiments. The collection bag frame could be improved further by adding in different-sized mesh layers to separate mosquitoes and other smaller or larger insects. Such a design would facilitate broad sampling of ‘vector species assemblages’ rather than simply targeting one species group as is the most commonly used approach. Using individual or blended semiochemical components that are attractive to multiple species groups is a challenging but achievable option for this^20^.

We have demonstrated that a 3D-printed trap provides comparable mosquito trapping efficacy in a temperate marshland habitat to two commercially available traps. The significant cost savings and flexibility afforded by the 3D-printed trap could accelerate the gathering of important entomological data worldwide.

## Methods

### Study sites

**Figure 2:**
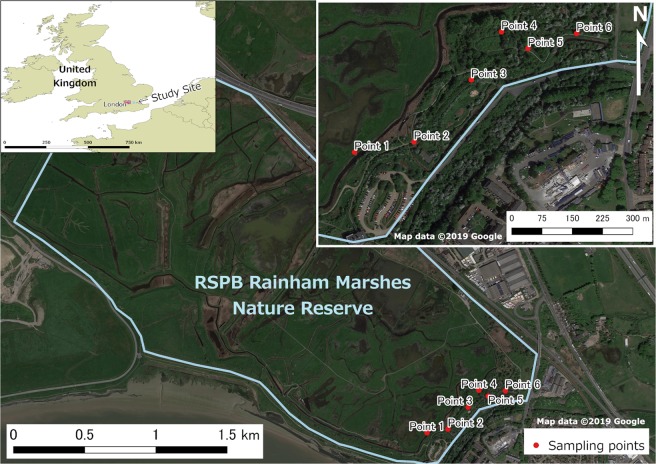
Study site and six sampling points in Essex, UK. The box on the top left shows the study site location within the UK, with the six sampling points magnified. Data from Natural Earth (https://www.naturalearthdata.com) was used to obtain country and county boundaries, and satellite images are from Google Maps (https://www.google.com/maps).

The study was conducted at RSPB Rainham Marshes Nature Reserve, Essex, UK (Grid ref: TQ547787; Fig. 2). The marshes are carefully managed with the aim of preserving populations of resident and migratory birds and other wildlife, in part through the grazing of approximately 100 head of cattle. Permanent water bodies and small diches can be found across the site throughout the year.

### Sampling strategy

**Figure 3:**
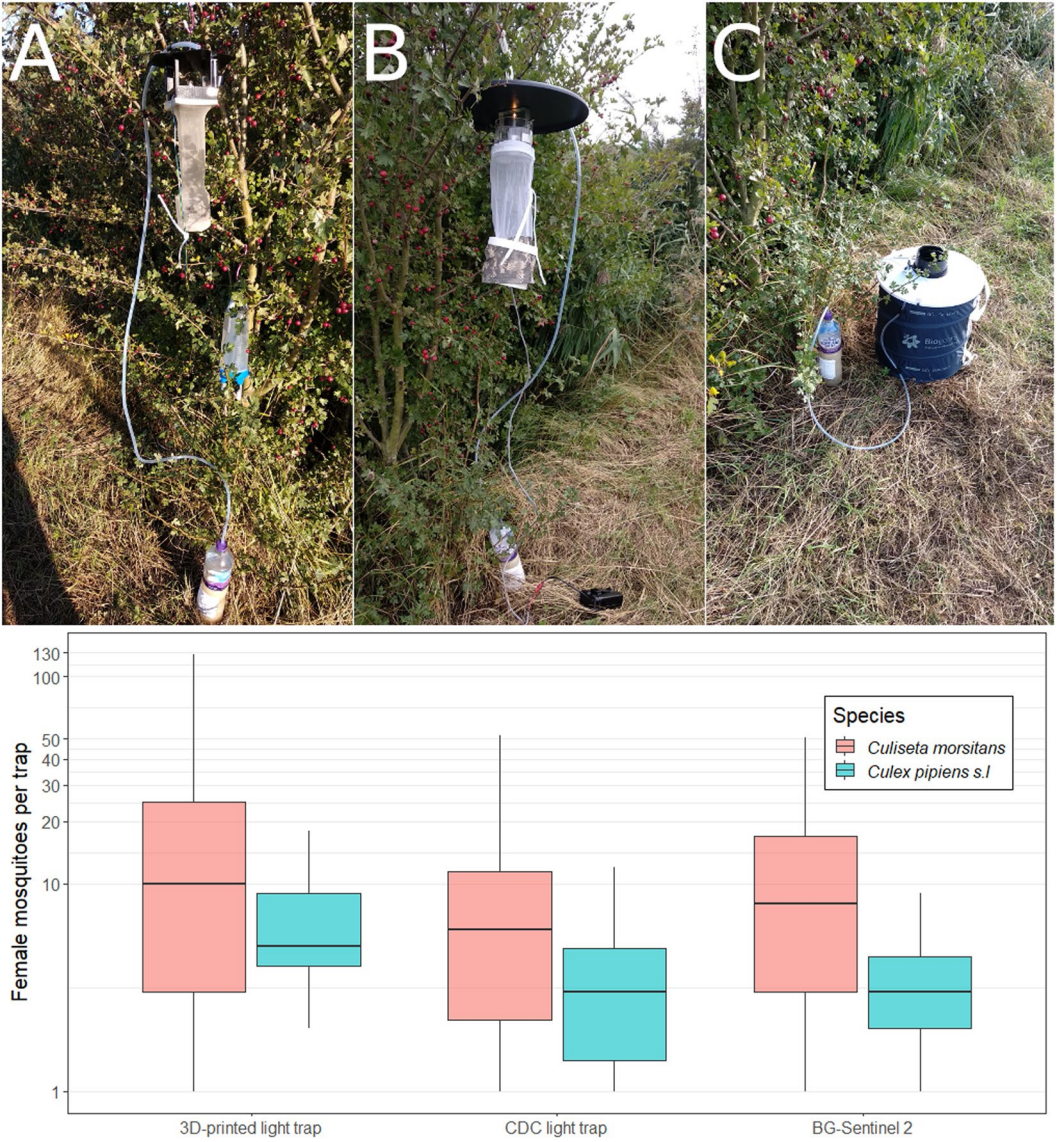
The three trap types, with CO_2_ bait, at sampling point 1: (**A**) 3D-printed trap; (**B**) CDC light trap; and (**C**) BG-Sentinel 2 trap. Each trap was connected to a yeast-mixture CO_2_ gas setup. The box plots represent the median and first and third quartiles of the main species of female mosquito collected in the three trap types. The y-axis is set to a log-scale.

A 6 × 6 Latin square design was used to compare mosquito sampling efficacy between two each of our 3D-printed mosquito light trap, the CDC light trap (model 512, John W. Hock, Gainsville, Florida, USA), and BG-Sentinel 2 trap (Biogents AG, Regensburg, Germany) at six sampling points (Fig. 2). The Latin square was created using a web application (https://hamsterandwheel.com/grids/index3d_shuffle_method.php). The experiment was conducted for two replicates of the Latin squire design (i.e., 12 nights) from the 5^th^ to 26^th^ of September 2018. All traps had a setup of a yeast-sugar-mixture (yeast 14 g; sugar 200 g, and water 1000 ml) to produce CO_2_ gas^21^, and only BG-Sentinel 2 traps had the attractant lure of artificial human skin scent (BG-Lure cartridge; Biogents AG, Regensburg, Germany). Approximately 1 hour prior to setting up the traps the yeast mixture was prepared in a 1.5 L PET bottle. To keep the bottle warm, one adhesive-backed disposable heat pad (Cura-Heat Back & Shoulder Pain, Kobayashi Health Care Europe, Ltd. UK) was attached onto the side of each bottle. A 1.8 m vinyl tube was run from the bottle to each trap, and the outlet of the tube was fixed using a hook and tape either on the top of light trap rain shield or beside the intake funnel of the BG-Sentinel 2 (Fig. 3). For the experiments, six sampling points approximately 50 m apart were set at the south eastern part of the marshes where trees are abundant (Fig. 2). The light traps were hung on a tree at 1.6 m above the ground level and BG-Sentinel 2 traps were placed on the ground. The batteries used for all traps were securely contained in a plastic case or plastic bag to avoid any direct contact with wild animals. The traps were operated from 16:00 until 09:00 the following morning.

### Trap design

A 3D model of the trap was designed and underwent preliminary testing in Nagasaki, Japan. To design the trap, 3D computer-aided design (CAD) software Design Spark Mechanical 2.0 was used (Electrocomponents plc Co., London, UK). A 3D printer was used to fabricate the trap components (I3 Mega; Shenzhen Anycubic Technology Co., Guangdong, China), using a black filament (Verbatim 55250, polylactic acid; Mitsubishi Chemical Media Co., Tokyo, Japan). The trap consisted of three 3D-printed pieces: a body-piece to hold a computer fan and two AA batteries to power the light bulb; a light-piece to hold a miniature light bulb; and a rain shield-piece for preventing any materials from entering the collection bag and to facilitate the attachment of a string to hang the trap (Fig. 1). An 8 × 8 cm computer case fan (F8 PWM; Arctic Co., Braunschweig, Germany) was inserted into the body-piece. To run the fan, a 5 V mobile battery (Anker PowerCore 20100; Anker Innovations Ltd., Shenzhen, China) with a USB step-up DCDC converter from 5 V to 12 V (generic brand available from https://www.aliexpress.com) was used. We installed electronic circuits using aluminium tape (Daiso Co., Hiroshima, Japan) to power a miniature light bulb (GA-10NH; Asahi Electronic Co., Osaka, Japan) with two 1.2 V AA rechargeable Nickel-Metal Hydride Batteries (BK-3HCD; Panasonic Co., Osaka, Japan) connected in parallel. At the bottom of the body-piece, we attached short stockings (Daiso Co., Hiroshima, Japan) and used a transparent cup (7.5 cm × 9.2 cm, 300 ml; 5–026–01; Sankoukasei Co., Osaka, Japan) as the collection cup. The material costs for the three parts printed in the UK was $3.78 USD (£2.9 GBP), and electronic parts were $6.84 USD (£5.27 GBP) for a total cost of $12.49 USD (£9.61 GBP) for the trap. In general, the battery for 25 hours operation (nearly 20,000 mhA) costs around $43 USD (£33 GBP).

### Mosquito identification

Sampled mosquitoes were pinned and morphologically identified to species level under a binocular microscope based on published keys^22,23^. The voucher specimens are maintained in the National History Museum in London.

### Statistical analysis

To test sampling efficacy between three kinds of traps, total and species-sex stratified numbers of mosquitoes were compared. Specifically, a Kruskal-Wallis rank sum test was performed after confirming the data was not normally distributed using the Shapiro–Wilk test. All statistical analyses (i.e. kruskal.test and shapiro.test commands) were performed using RStudio (version 1.1.447, 64 bit) with R (version 3.4.1, 64 bit) backend. The statistical significance level was set to be less or equal to 0.05. In addition, QGIS (version 3.2.2, 64 bit) was used to create the study site map.

### Third party rights

We created map using data from Natural Earth and Google Maps. Those allow free use of their maps for research purposes with acknowledgement.

## Supplementary information

Supplementary Figures

Supplementary Data 1

Supplementary Data 2

Supplementary Data 3

Supplementary Dataset S1

## Data Availability

All data collected in the study is enclosed in the manuscript.
