# Supplementary material for: Field testing of a lightweight, inexpensive, and customisable 3D-printed mosquito light trap in the UK
Source: Sci Rep. 2019 Aug 6;9:11412. doi: 10.1038/s41598-019-47511-y (PMC6684613; doi:10.1038/s41598-019-47511-y)
Supplement: Supplementary file 1 — Supplementary Figures [file 41598_2019_47511_MOESM1_ESM.docx]

**Field testing of a lightweight, inexpensive, and customisable 3D-printed mosquito light trap in the UK**

Tomonori Hoshi^1,2,3*^, Victor A. Brugman^1,4^, Shigeharu Sato^3,5^, Thomas Ant^1^, Bumpei Tojo^3^, Gaku Masuda^3^, Satoshi Kaneko^2,3^, Kazuhiko Moji^2,3^, Jolyon M. Medlock^6^, James G. Logan^1^

1) London School of Hygiene and Tropical Medicine, Keppel Street, London, WC1E 7HT, United Kingdom

2) Department of Eco-Epidemiology, Institute of Tropical Medicine, Nagasaki University, Nagasaki, 852-8523, Japan.

3) School of Tropical Medicine and Global Health, Nagasaki University, Nagasaki, 852-8523, Japan.

4) Vecotech Ltd, Keppel Street, London, WC1E 7HT, United Kingdom

5) Faculty of Medicine and Health Sciences, University Malaysia Sabah, Sabah, 88400, Malaysia

6) Public Health England, Porton Down, Salisbury, SP4 0JG, United Kingdom

*Corresponding author: Tomonori Hoshi (tomonori.hoshi.japan@gmail.com)


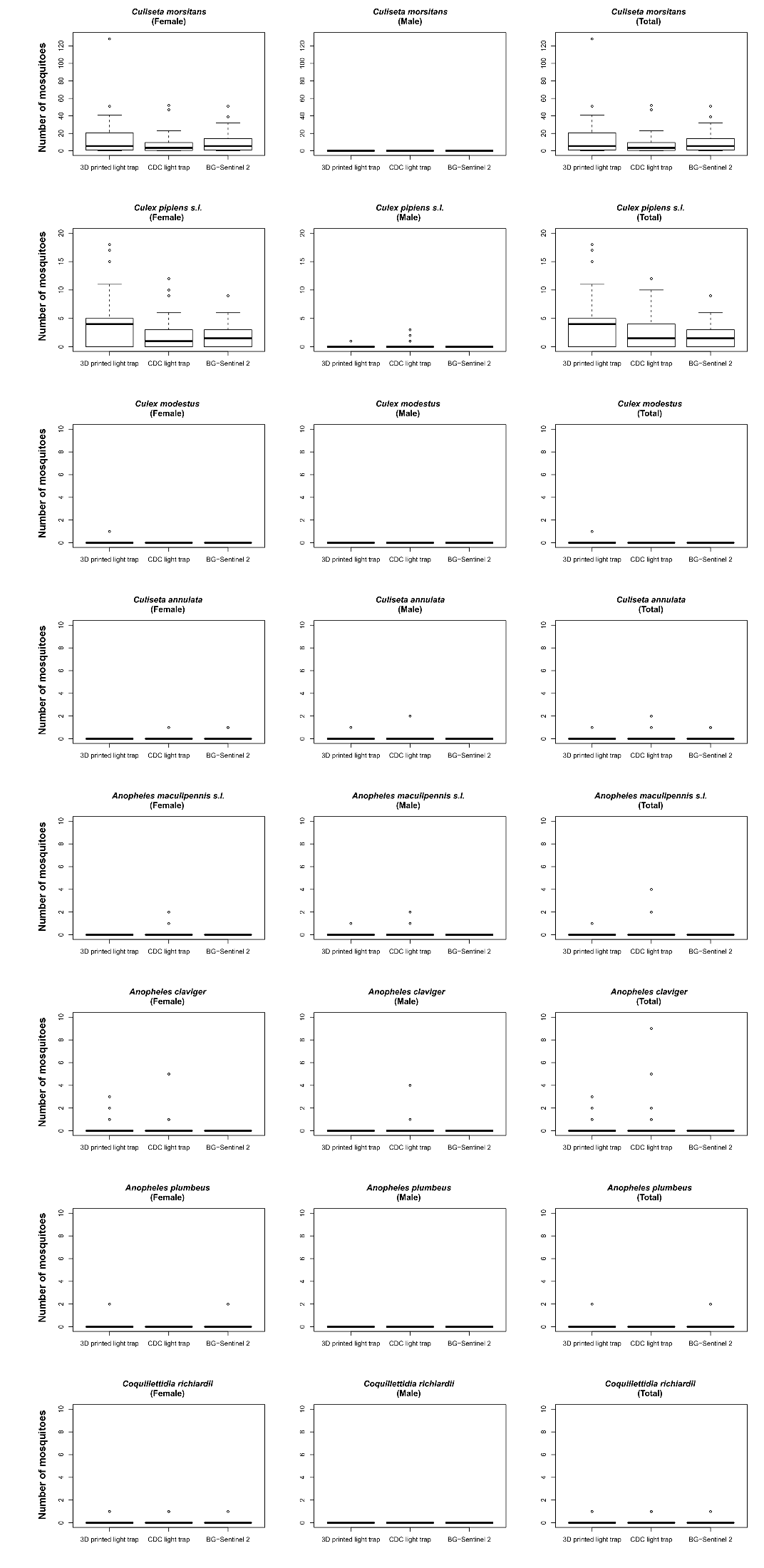


Supplementary Figure S1. Box plots show the variance of the total and species-sex stratified number of mosquitoes by sampling methods.


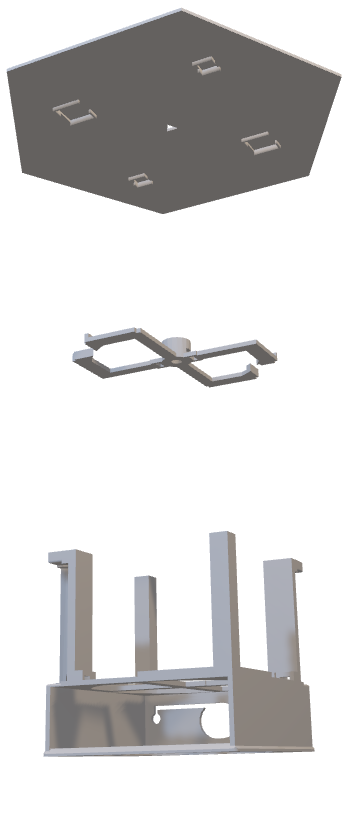

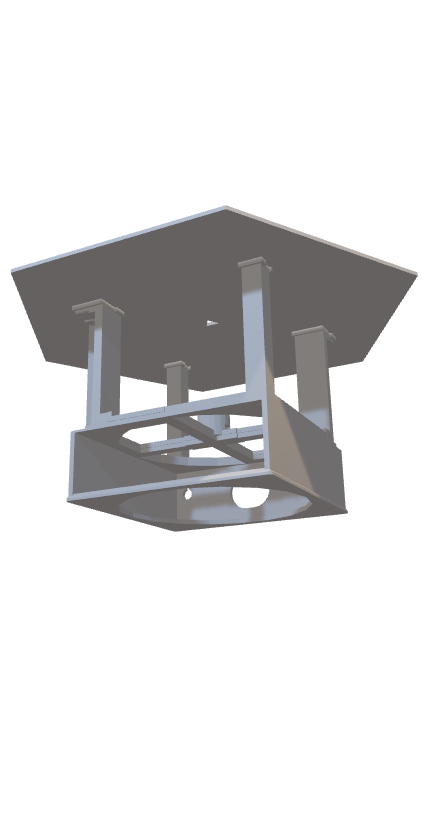


B

A

Supplementary Figure S2. Tested 3D-printed trap model. Three pieces of 3D-printed parts (Rain Shield, Light, and Body frame) are assembled in the panel A and disassembled in the panel B. The models can be rotated along three different axes.


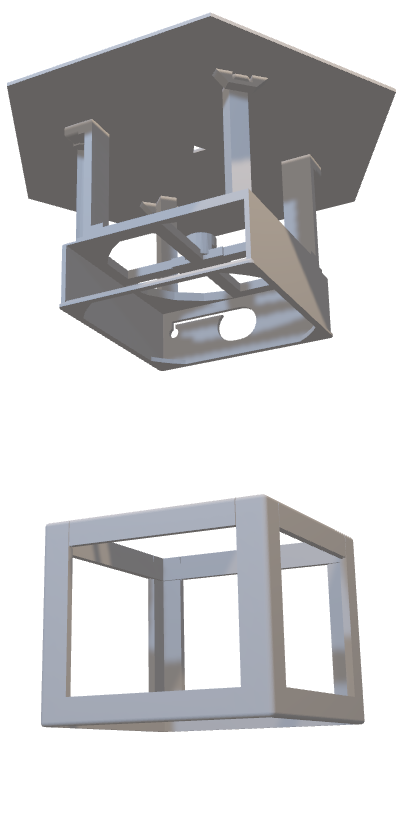

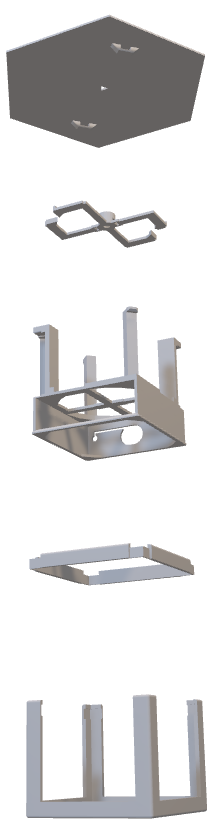


B

A

Supplementary Figure S3. Improved 3D-printed trap model. Five pieces of 3D-printed parts (Rain Shield, Light, Body frame, and Collection box frame) are assembled in the panel A and disassembled in the panel B. The models can be rotated along three different axes.
