# Supplementary material for: Field testing of a lightweight, inexpensive, and customisable 3D-printed mosquito light trap in the UK
Source: Sci Rep. 2019 Aug 6;9:11412. doi: 10.1038/s41598-019-47511-y (PMC6684613; doi:10.1038/s41598-019-47511-y)
Supplement: Supplementary file 2 — Supplementary Data 1 [file 41598_2019_47511_MOESM2_ESM.zip › Supplementary Dataset 1/Readme.docx]

**Field testing of a lightweight, inexpensive, and customisable 3D-printed mosquito light trap in the UK**

Tomonori Hoshi^1,2,3*^, Victor A. Brugman^1,4^, Shigeharu Sato^3,5^, Thomas Ant^1^, Bumpei Tojo^3^, Gaku Masuda^3^, Satoshi Kaneko^2,3^, Kazuhiko Moji^2,3^, Jolyon M. Medlock^6^, James G. Logan^1^

1) London School of Hygiene and Tropical Medicine, Keppel Street, London, WC1E 7HT, United Kingdom

2) Department of Eco-Epidemiology, Institute of Tropical Medicine, Nagasaki University, Nagasaki, 852-8523, Japan.

3) School of Tropical Medicine and Global Health, Nagasaki University, Nagasaki, 852-8523, Japan.

4) Vecotech Ltd, Keppel Street, London, WC1E 7HT, United Kingdom

5) Faculty of Medicine and Health Sciences, University Malaysia Sabah, Sabah, 88400, Malaysia

6) Public Health England, Porton Down, Salisbury, SP4 0JG, United Kingdom

*Corresponding author: Tomonori Hoshi (tomonori.hoshi.japan@gmail.com)

**Readme**

This document explains how to use *Supplementary Info 1* to create the 3D-printed trap.

**Folder structure**

*Supplementary Data 1* contains “*Tested model*” and “*Improved model*” folders, and this Readme document. The former folder has trap modelling data tested in this study, and the latter folder contains the improved design based on the authors' experiences in the field in Japan and the UK. Both models are available, but it is highly advisable to use the improved model, and this document describes how to build the improved model.

In each folder, there are four subfolders:

1. *gcode*
2. *3mf*
3. *stl*
4. *Cura 3.6 settings*

These will be explained in the document below.

**Print**

Depending on your printer settings and experience, there are three options for use indicated below.

**Use authors’ printer settings**

Users who have the same model of the 3D printer used in this study, Anycubic I3 Mega, can simply transfer all gcode files in the *1. gcode* folder onto an SD card and follow the manufacturer’s instructions of the printer to fabricate all gcode files. After printing all pieces, they are ready to assemble together as described in **Assembly**. The gcode files have the authors’ settings for printing (i.e. filament type and temperature, etc), but if the settings do not work in your printing environments, it is possible to adjust the settings as indicated below.

**Users who need to adjust printer settings**

The printing settings of gcode files cannot be edited. Therefore, to change the settings, open all 3mf files in *2. 3mf folder* using Cura 3.6. While opening the files, Cura 3.6 asks how to open the file: “Open as a project” or “Import models”. Select the former option and open the file. Now the software shows the authors’ printing settings on the right-side panel of the software window. In the panel, it is possible to adjust printer settings. After finishing the adjustment, press “Save to file” button on the right corner at the bottom, which exports a gcode file having the adjusted settings. This process needs to be repeated for each trap piece.

**Advanced users with different printer models**

Some users may need stl format files (i.e. raw modelling data exported from CAD software) to process using their preferable software. For those users, all stl files are available in *3. stl* folder. In addition, the authors' printer setting files for each trap piece can be found in *4. Cura 3.6 settings* folder, which can be imported to Cura 3.6 and the details of the settings checked.

**Assembly**

**Installation of electronic circuits**

Aluminium tape or relevant conductive material of the following sizes of 4.5—5 mm wide strips are required:

- Two 9 cm strips … Install into the blue highlighted places of the light piece (a).
- One 17 cm strip … Install into the red highlighted groove of the body piece (b).
-
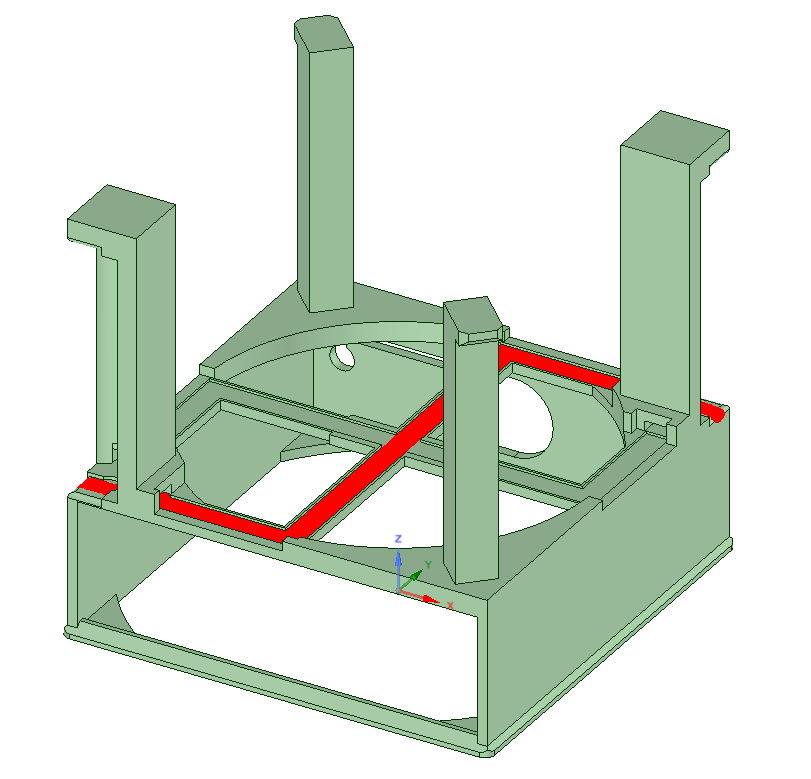

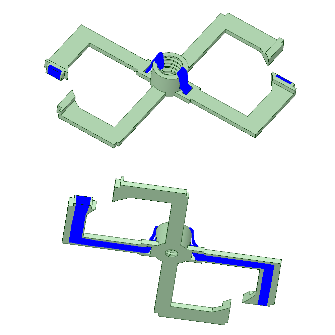

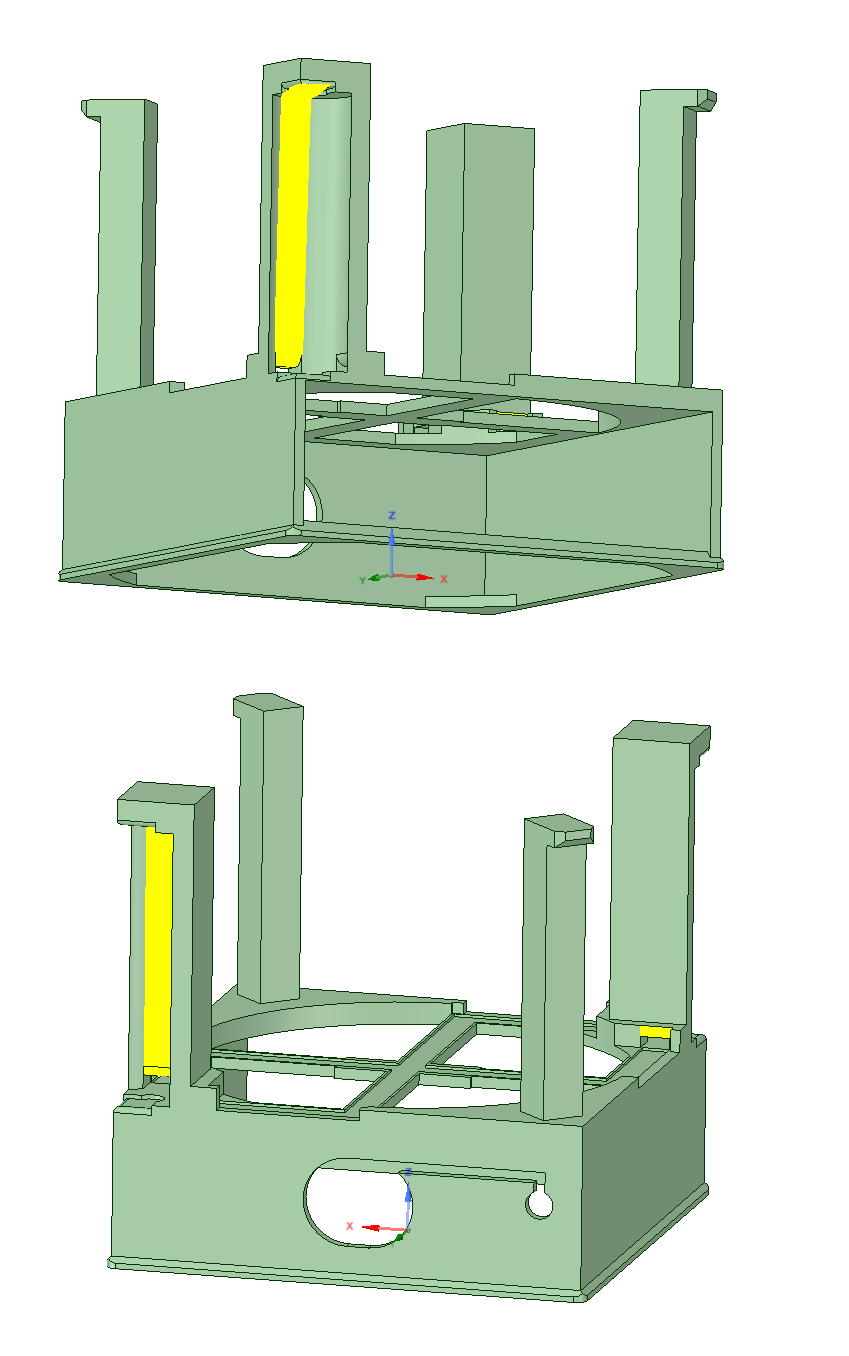
Two 7 cm strips … Install into the yellow highlighted places of the body piece (c).

(c)

(b)

(a)

There may be a gap between the light and the body pieces once assembled. To ensure a complete circuit, any gaps can be filled with further pieces of aluminium tape or foil.

**Assembly of all pieces**

To assemble and operate the 3D-printed trap, following electronic parts are necessary.

1. One 1.5v miniature bulb
2. One 8 x 8 cm computer fan
3. One USB step up converter from 5v to 12v^[[1]](#footnote-1)^
4. One 15 cm string
5. One short stockings

1. Put the light piece on the body piece. These two pieces are designed to fit tightly, so may require a little force to clip together.


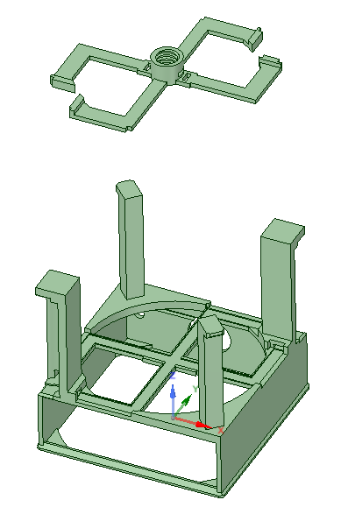


2. Screw the miniature bulb (d) onto the centre socket of the light piece.

3. Insert the computer fan (e) into the room of the body underneath the light. When inserting the fan, make sure the electronic wire passes through the hole at the back of the body piece. Strip the tip of the wires, (e) and (f). Then, connect the positive wires of the tips of (e) and (f), and likewise the negative wires by twisting the wire together, crimping the wires, or soldering the wires.


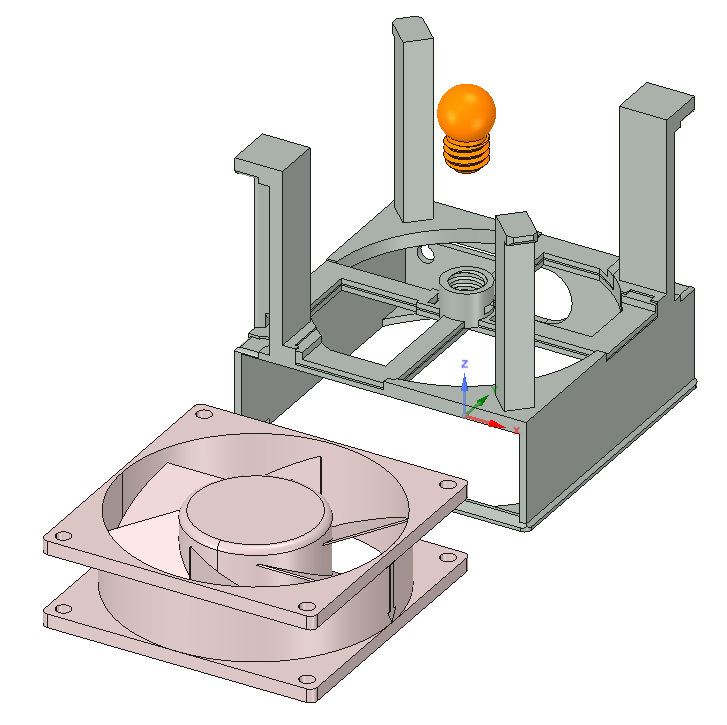


(e)

(d)


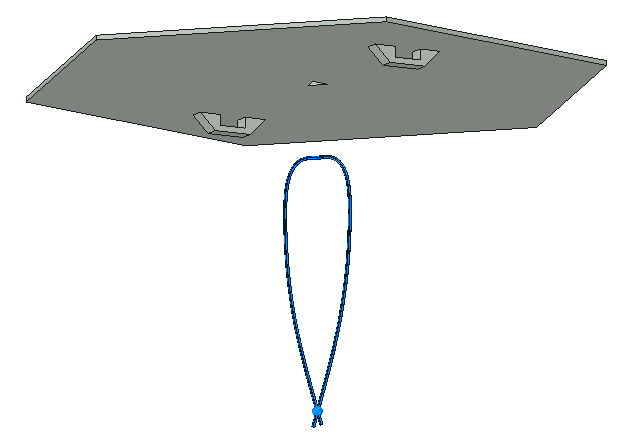
4. Fold the 15 cm string (g), and tie the ends up to make a knot. The size of the knot needs to be larger than the centre hole in the rain shield. Now the string can be installed in the centre hole. Pass through the folded edge of (g) from the underside to the topside of the rain shield piece.

(g)

5. The rain shield can be attached to the body segment. Two slits on the rain shield provide additional support to hold the two struts of the body.


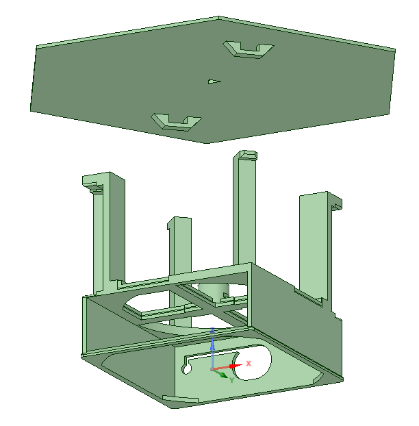


6. Put the square piece of the upper collection bag frame piece on the lower piece of the collection bag frame. Four corners of the upper piece can fit into the grooves of the four poles of the lower piece.


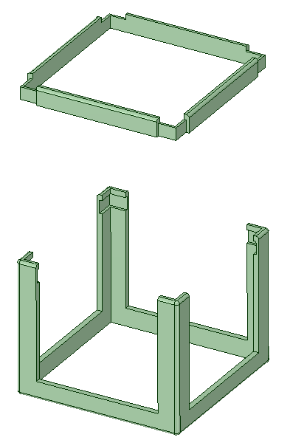


7. Put the assembled collection bag frame into a short stocking (h).


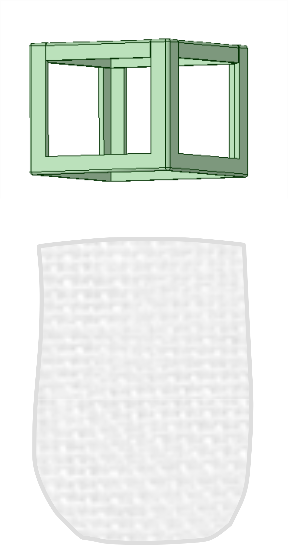


(h)

**Operation of the trap**

To operate the trap, the following two items are needed.

(i) Two AA batteries

(j) One power bank battery (preferably >10,000mAh for overnight operation)


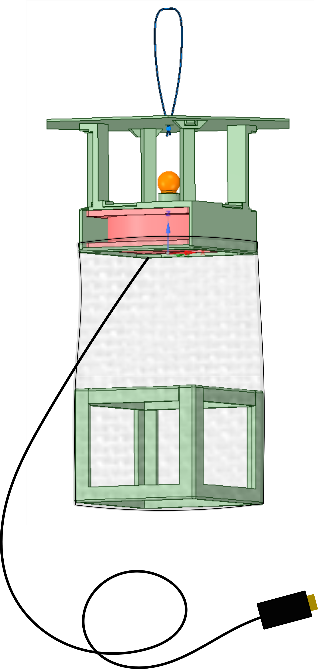


(i)

(i)

(j)

1. Insert one of the two AA batteries (i) to each battery socket on the pole. Once the batteries are inserted the light bulb will turn on.

2. Connect a power bank battery (j) to the USB step up converter (f). Once the power bank has been connected the fan will start and the trap is fully operational.

To carry the trap, the collection bag frame can fit into between the body and rain shield. A short stocking (h) needs to be removed to prevent from tearing.


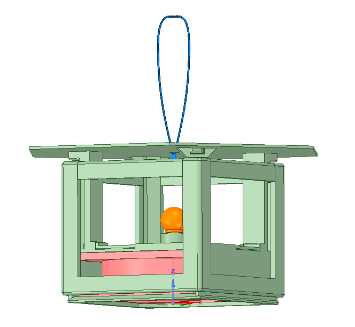

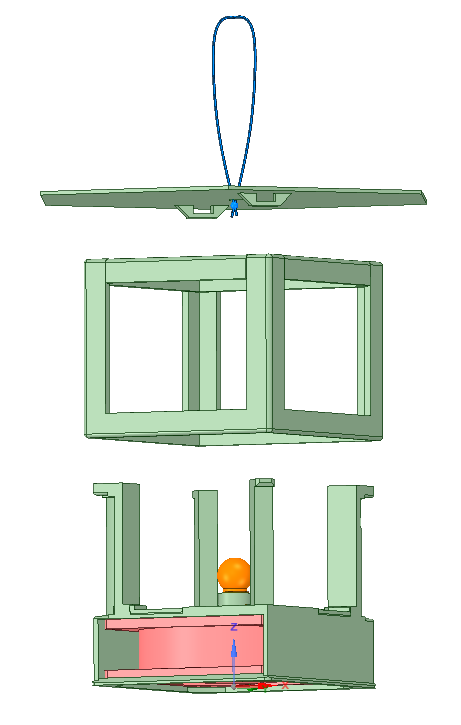


1. The USB step up converter is a transformer to increase the voltage from 5v to 12v, the required voltage to operate a computer fan. [↑](#footnote-ref-1)
